# Antibody responses to *Plasmodium falciparum* and *Plasmodium vivax* blood-stage and sporozoite antigens in the postpartum period

**DOI:** 10.1038/srep32159

**Published:** 2016-08-25

**Authors:** Alistair R. D. McLean, Machteld E. Boel, Rose McGready, Ricardo Ataide, Damien Drew, Takafumi Tsuboi, James G. Beeson, François Nosten, Julie A. Simpson, Freya J. I. Fowkes

**Affiliations:** 1 Macfarlane Burnet Institute of Medical Research, Melbourne 3004, Australia; 2 Centre for Epidemiology and Biostatistics, Melbourne School of Population and Global Health, The University of Melbourne, Melbourne 3004, Australia; 3 Shoklo Malaria Research Unit (SMRU), Mahidol-Oxford Tropical Medicine Research Unit, Faculty of Tropical Medicine, Mahidol University, Mae Sot 63110, Thailand; 4 Centre for Tropical Medicine and Global Health, Nuffield Department of Medicine, University of Oxford, Oxford OX1 2JD, UK; 5 Division of Malaria Research, Proteo-Science Center, Ehime University, Matsuyuma 790-8577, Japan; 6 Department of Microbiology, Monash University 3800, Australia; 7 Department of Epidemiology and Preventative Medicine, Monash University 3800, Victoria, Australia

## Abstract

During pregnancy a variety of immunological changes occur to accommodate the fetus. It is unknown whether these changes continue to affect humoral immunity postpartum or how quickly they resolve. IgG levels were measured to *P. falciparum* and *P. vivax* antigens in 201 postpartum and 201 controls over 12 weeks. Linear mixed-effects models assessed antibody maintenance over time and the effect of microscopically confirmed *Plasmodium* spp. infection on antibody levels, and whether this was different in postpartum women compared with control women. Postpartum women had reduced *Plasmodium* spp. antibody levels compared to controls at baseline. Over 12 weeks, mean antibody levels in postpartum women increased to levels observed in control women. Microscopically confirmed *P. falciparum* and *P. vivax* infections during follow-up were associated with an increase in species-specific antibodies with similar magnitudes of boosting observed in postpartum and control women. Antibodies specific for pregnancy-associated, VAR2CSA-expressing parasites did not rapidly decline postpartum and did not boost in response to infection in either postpartum or control women. After pregnancy, levels of malaria-specific antibodies were reduced, but recovered to levels seen in control women. There was no evidence of an impaired ability to mount a boosting response in postpartum women.

Malaria in pregnancy, caused by *Plasmodium falciparum* and *Plasmodium vivax*, is a major global public health problem. Over 125 million pregnancies are at risk of malaria annually^1^. Pregnant women are at increased risk of infection with negative consequences for the mother and baby including maternal anaemia, low birth weight and neonatal mortality^2^. The increased risk of *P. falciparum* and *P. vivax* infection during pregnancy may be partly due to immunological changes to accommodate the fetus during pregnancy^3^. The increased risk of *P. falciparum* infection can also be attributed to the ability of *P. falciparum* infected erythrocytes to bind and sequester in the placenta^4^,^5^, a pathology that does not occur often in *P. vivax* infections^6^. Antibody-mediated immunity against *P. falciparum* variants that sequester during pregnancy develops over successive pregnancies, conferring a degree of protection in future pregnancies^6^. Postpartum women may also be at differential risk of *P. falciparum*^7^,^8^,^9^ and *P. vivax*^9^ infection compared to non-postpartum women. This suggests that the altered risk of malaria during pregnancy may continue after delivery, however, little is known about humoral immunity to malaria in the postpartum period.

Individuals living in malaria endemic areas develop naturally acquired immunity to *Plasmodium* spp. infections with repeated infections^10^. Naturally acquired antibodies to sporozoites can protect against liver-stage infection and antibodies targeting blood-stage antigens can suppress high parasite densities and progression to symptomatic disease^10^. Breadth and magnitude of antibody responses are important, with responses to a repertoire of antigens associated with increased protection against disease^11^,^12^,^13^,^14^, while also acting as biomarkers of past exposure^15^.

Pregnant women in malaria endemic settings may possess antibodies to a broad range of antigens but are still susceptible to *P. falciparum* and *P. vivax* infection in pregnancy^16^. In the case of *P. falciparum*, this is largely because they lack antibodies to placental binding *P. falciparum* isolates that express VAR2CSA, a variant surface antigen expressed on the surface of the infected erythrocyte involved in placental sequestration^17^,^18^. As VAR2CSA is primarily encountered by the immune system during pregnancy, VAR2CSA antibodies are typically absent or at low levels prior to first pregnancy and increase with exposure through multiple pregnancies^19^. Antibody responses to other blood-stage antigens may also boost upon exposure in pregnancy^20^. There is currently a lack of consensus as to how antibody responses, particularly VAR2CSA antibodies, are maintained or decline postpartum. Previous studies have reported increases^21^,^22^, no change^23^,^24^ and decreases^22^,^23^,^24^,^25^,^26^ in *P. falciparum* antibody levels postpartum compared to pregnancy depending on study site and antigen. However, no study included a control group of non-pregnant women to enable comparisons, nor had available data on the presence of *Plasmodium* infection between serological measurements^21^,^22^,^23^,^24^,^25^,^26^. Importantly, most studies assessed antibodies at one time-point postpartum^21^,^22^,^23^,^24^,^26^, thereby not taking into account potential fluctuations and limiting inferences about postpartum antibody persistence. Additionally, past research has only considered *P. falciparum* targets in African settings.

To address the paucity of data and investigate whether the postpartum period comprises a period of humoral immunity transitioning to a non-pregnant state, we determined *P. falciparum* and *P. vivax* antibody levels at multiple time points in postpartum and control women living at the Thai-Myanmar border. We sought to determine whether there are differences in boosting and maintenance of *Plasmodium* spp. specific antibodies in postpartum women compared with control women.

## Methods

### Ethics statement

The study was performed in accordance with the guidelines approved by The Alfred Hospital Human Research and Ethics Committee, Melbourne, Australia (88/13); The Faculty of Tropical Medicine Ethics Committee, Mahidol University Bangkok, Thailand (MUTM 2007-023) and Oxford Tropical Medicine Ethical Committee, Oxford University, England (Code 002-07). All participants gave informed consent prior to enrolment.

### Study design and population

This study investigated 201 postpartum cases and 201 non-postpartum (and non-pregnant) controls over a 12-week period of study. Individuals with at least 3 sera samples available, or who experienced a microscopically confirmed *P. falciparum* infection, were selected from a larger case-control study of malaria in the postpartum period (described previously^9^). Briefly, pregnant women attending Shoklo Malaria Research Unit (SMRU) antenatal clinics located in North-Western Thailand from November 2007 to September 2009 were invited to participate and were asked to find a non-pregnant female of similar age and location to act as a control. During the follow-up period all women were tested for the presence of *Plasmodium* infection via weekly light microscopy of blood smears and completed questionnaires on behavior. Monthly blood samples (~200 μl) were collected to assess haematocrit and serology. The first measurement (baseline) was obtained at first postpartum visit (median days since delivery: 13, interquartile range: 9–16). Microscopically confirmed *P. falciparum* infections were treated with mefloquine and artesunate. Microscopically confirmed *P. vivax* infections were treated with chloroquine.

### Antibody determination

We measured antibodies to eight *P. falciparum* antigens (merozoite (*Pf*EBA140_RIII-V_, *Pf*EBA175_RII_, *Pf*EBA175R_III-V_, *Pf*AMA-1, *Pf*MSP2, *Pf*Rh2); *P. falciparum* infected erythrocyte: *Pf*DBLα; sporozoite (*Pf*CSP), all 3D7 alleles) and four *P. vivax* antigens (merozoite (*Pv*AMA-1 (Palo Alto), *Pv*MSP1_19_ (SalI), *Pv*DBP (SalI)); sporozoite (*Pv*CSP (Belem and PNG)) and one VAR2CSA expressing *P. falciparum* parasite strain (CS2)^27^ (Supplementary Table 1). These antibodies represent different lifecycle stages and are biomarkers of exposure and protective immunity^28^,^29^.

1462 sera samples from 402 women were assayed by high-throughput ELISA and flow cytometry. Nine seroreactive pools from Thailand and Papua New Guinea individuals were included as positive controls for standardisation and 37 non-exposed Melbourne donors acted as negative controls. ELISA was conducted as described previously^20^. The locations of samples on plates were randomised across the entire cohort and for each assay all samples were processed on the same day to minimise batch effects. Reactivity from the nine positive controls (in quadruplicate) was used to adjust for any inter-plate batch variability. Antigen and sera concentration are provided in Supplementary Table 1. Testing for IgG binding to the surfaces of CS2 infected erythrocytes was conducted as described previously^30^. Data were acquired by flow cytometry (FACSVerse, BD Biosciences) and analysed using FlowJo (FlowJo LLC, OR, USA). Assay output was derived by subtracting the mean fluorescence intensity (MFI) of uninfected erythrocytes from the MFI of trophozoite-infected erythrocytes. Seropositivity was defined as an OD or MFI exceeding the mean plus three standard deviations of negative controls.

### Statistical analysis

Statistical analyses were performed using Stata Version 13.1 (StataCorp, College Station, TX, USA). Correlations of antibodies at baseline were assessed using Spearman’s rank correlation coefficients. Antibody levels were analysed as log transformed continuous variables centred on the median value (log_2_([OD or MFI] + 0.001) − median(log transformed value)). Overall *P. falciparum* and *P. vivax* merozoite immunity scores were derived by calculating the average of the log transformed (centred) values for each antibody targeting species-specific merozoite antigens. To investigate if postpartum status was associated with species-specific antibody responses, linear mixed-effects modeling (with random intercept and slope) of log_2_ antibody levels versus time was performed with interaction terms between postpartum status and time (weeks). This enabled us to compare the slope of antibodies over time in postpartum women with control women.

Potential confounders, selected *a priori* using a causal diagram^30^, included clinic attended (Mawker Thai/Wang Pha/Walley/Mu Ler Chai), age (years) and history of working outdoors. In the case of *Pf*VAR2CSA expressing parasites, a binary variable of gravidity ≥3 (note, gravidity of 0, 1 and 2 had similar *Pf*VAR2CSA antibody levels) was included as a confounder instead of age, as gravidity is more predictive than age for placenta-adherent parasite immunity, and age and gravidity were highly collinear. In a subgroup analysis of postpartum women, detectable (by light microscopy) species-specific infection during pregnancy (yes/no) was included as a covariate. To assess the impact of changing haematocrit on antibodies over time, an interaction term between time and haematocrit change (≥4%) was assessed. To assess whether antibodies were boosted in response to infection, species-specific infection during follow-up was included as a time-varying variable with presence of infection (yes or no) at any visit prior to the corresponding antibody level measurement. The effect of heterologous species infection was assessed for each antibody response but only incorporated into the final model if p < 0.05. Given that we have reported all statistical comparisons conducted, p values were presented unadjusted for multiple comparisons^31^. Associations were interpreted based on the magnitude and direction of effect in addition to confidence intervals and p values.

## Results

### Characteristics of postpartum and control women

This study comprised 201 postpartum women and 201 controls living in North West Thailand (Table 1). Bednet use was high in both postpartum and control women (>93%); postpartum women were less likely to report a recent history of working outdoors than control women (60% versus 72%). During follow-up, 22 postpartum and 32 control women experienced a microscopically confirmed *P. falciparum* infection; 67 postpartum and 50 control women experienced a microscopically confirmed *P. vivax* infection. One postpartum woman and four control women experienced a co-infection.

### *P. falciparum* and *P. vivax* antibodies in postpartum and control women at baseline

**Figure 1:**
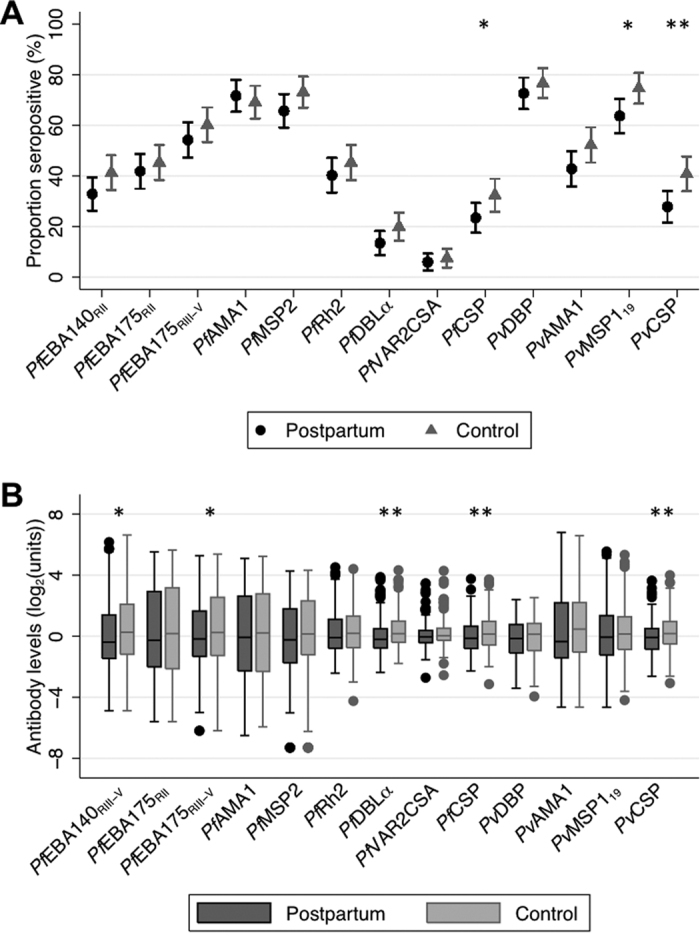
Antibodies to *Plasmodium* species antigens at baseline. Antibody levels were determined in all available postpartum women (n = 201) and control women (n = 201) (**A)** Seroprevalence against *P. falciparum* and *P. vivax* amongst postpartum (black circles) and control women (grey triangles). Bars indicate 95% confidence intervals. (**B)** Box and whiskers plots of IgG levels (log_2_(MFI) for PfVAR2CSA, log_2_(OD) for all other antibodies) against *P. falciparum* and *P. vivax* antigens amongst postpartum (black) and control (grey) women. Horizontal lines in box indicates median, box indicates the interquartile range, whiskers indicate the highest and lowest values within 1.5*interquartile range of the first and third quartiles, dots represent outliers. A single asterix denotes p < 0.05, a double asterix denotes p < 0.01 from Wilcoxon rank-sum and chi square tests.

At baseline, seroprevalence and levels of antibodies against *P. falciparum* and *P. vivax* antigens were reduced in postpartum compared with control women, with varying degrees of magnitude depending on antigen (Fig. 1). Median (interquartile range) seroprevalence of the antibodies investigated was 15% (9%, 25%) lower in control women than postpartum women. Antibody levels against all *Plasmodium* spp. targets investigated were positively correlated with each other similarly in both postpartum and control women (median (IQR) pairwise correlations 0.40 (0.29, 0.48) and 0.42 (0.33, 0.53) respectively). In all women, antibody responses specific for *P. falciparum* merozoite antigens were more strongly correlated with each other (median: 0.65 (IQR: 0.60, 0.71)) than other *P. falciparum* antigens (*Pf*CSP, *Pf*DBLα and *Pf*VAR2CSA). The homologous antigen pairs *Pf*CSP/*Pv*CSP (correlation of 0.67) and *Pf*AMA1/*Pv*AMA1 (correlation of 0.44) displayed the strongest levels of across-species association.

### Antibody dynamics in postpartum and control women

**Figure 2:**
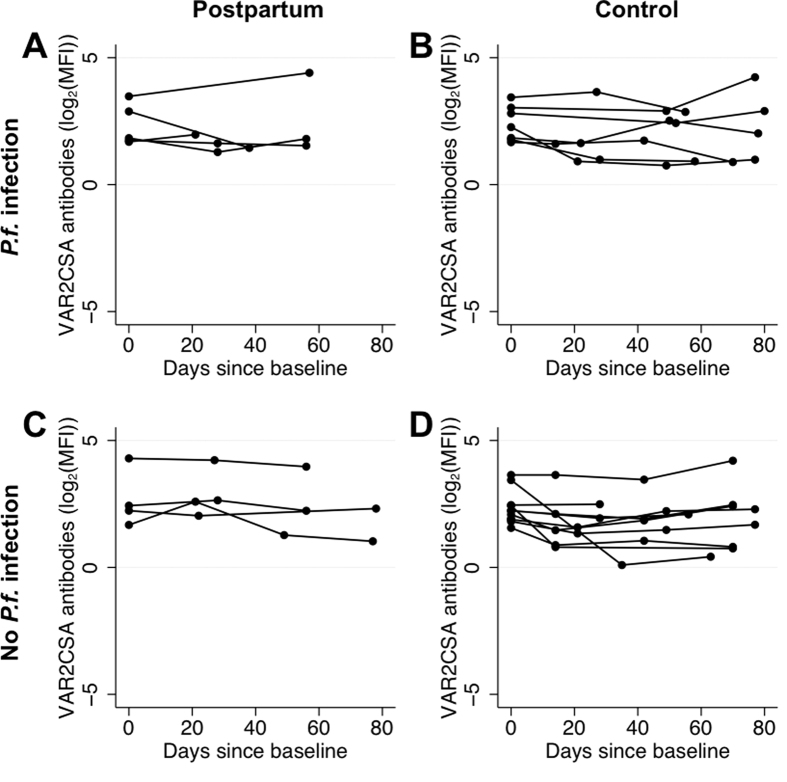
VAR2CSA antibodies (log_2_(MFI)) over time in women seropositive at baseline. Levels of VAR2CSA antibodies (log_2_(MFI)) over time in (**A**) postpartum women with a *P. falciparum* infection detected post-delivery, (**B**) control women with a *P. falciparum* infection detected, (**C**) postpartum women without any *P. falciparum* infection detected post-delivery and (**D**) control women without any *P. falciparum* infection. Each individual woman’s VAR2CSA antibodies over time are represented by a series of connected dots.

Availability of multiple serological measurements for each woman allowed us to assess postpartum antibody dynamics and relate these findings to control women. Due to the large number of merozoite responses determined and the considerable collinearity between merozoite responses, an average *P. falciparum* merozoite response and *P. vivax* merozoite response were generated for ease of interpretation. In the absence of an infection, *P. falciparum* and *P. vivax* antibody levels appeared relatively stable in postpartum and control women during the 12-week follow-up period (Supplementary Figures 1 and 2). Residual variances were significantly lower in those who did not experience species-specific infection than in those who did experience species-specific infection (likelihood-ratio test, all p < 0.001, except *Pf*VAR2CSA p = 0.05). Many postpartum and control women who were *Pf*VAR2CSA seropositive at baseline maintained their responses over the course of the study (Fig. 2). *Pf*VAR2CSA seropositive women with a documented infection during pregnancy had a significant negative slope of antibodies over time (−0.06 (95% Confidence Interval: −0.11, −0.02; p = 0.01) while in the other *Pf*VAR2CSA seropositive women there was no significant decline in antibodies (p = 0.50).

In order to assess differences in antibody responses at baseline and over time in postpartum and control women, multivariable linear mixed-effects models were constructed for each antibody response (Table 2; Supplementary Tables 2 and 3). Age and a history of working outdoors, both indicators of past exposure, were associated with increased antibody levels to *P. falciparum* and *P. vivax* targets. Adjusted mean levels of antibodies to *Pf*VAR2CSA expressing parasites were 0.29 (95% CI 0.15–0.42) higher in women with at least three prior pregnancies compared to those with two or fewer prior pregnancies (Table 2, p < 0.001). At baseline, postpartum women had lower mean levels of *P. falciparum* and *P. vivax* antibodies targeting merozoites, sporozoites and infected erythrocytes compared to control women (adjusted mean difference 0.16 to 0.32 (Table 2)).

**Figure 3:**
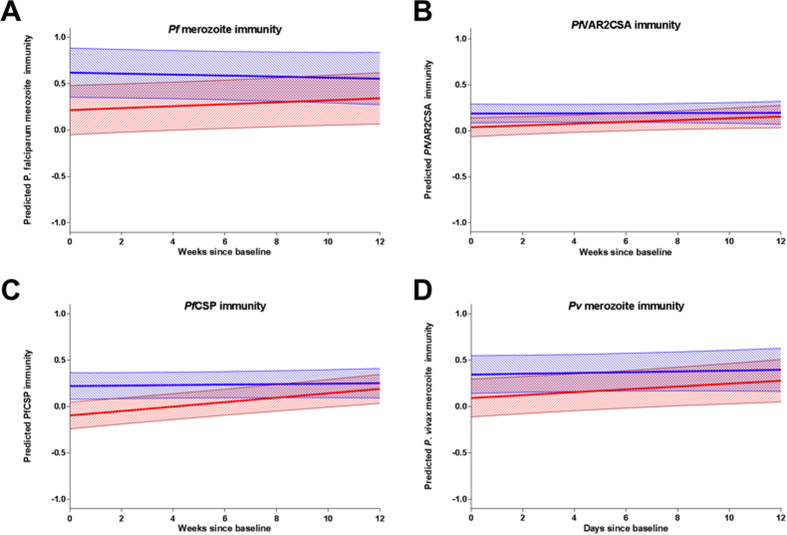
Predicted mean antibody level trajectories over 12 weeks follow-up for postpartum and non-pregnant controls. Mean predicted values (line) and 95% confidence intervals (shading) of antibody reactivity to (**A**) *Pf* merozoite, (**B**) *Pf*VAR2CSA, (**C**) *Pf*CSP, (**D**) *Pv* merozoite (log_2_(MFI) for *Pf*VAR2CSA and log_2_(OD) for all others) are plotted for postpartum (red) and non-pregnant control (blue) women. There was strong evidence for an interaction between postpartum and time for *Pf*CSP antibodies (likelihood-ratio test p value for interaction between postpartum and time) (*Pf*CSP p < 0.001), moderate evidence for *Pf* merozoite immunity (p = 0.09), weak evidence for *Pv* merozoite immunity (p = 0.25) and *Pf*VAR2CSA (p = 0.19).

Mean antibody levels did not change significantly over time in control women (Table 2, all p > 0.14). In contrast, the mean level of antibodies in postpartum women increased during the follow-up period; with a 0.01–0.03 mean increase per week follow-up (Table 2). As such, over the course of the 12 weeks of follow-up the initial difference in merozoite, sporozoite and infected erythrocyte antibody levels between postpartum and control women reduced over time as mean antibody levels of postpartum and control women converged (Fig. 3). Adjusting for haematocrit in the models did not alter these observations. Similarly, in a subset analysis of postpartum women accounting for species-specific infections during pregnancy (which were associated with increased baseline antibody levels), mean postpartum antibody levels increased with time (p = 0.10 for *Pf* merozoite immunity, p < 0.05 for all others) (Supplementary Table 4).

### The effect of species-specific infection on *P. falciparum* and *P. vivax* antibody levels in postpartum and control women

To investigate the effect of species-specific infection on antibody levels, and whether postpartum status modified this effect, the previous models were expanded to incorporate species-specific infection, detected by light microscopy, during follow-up as a time-varying variable (Table 3 and Supplementary Table 5). There was strong evidence of boosting with species-specific infection in antibody responses to *Pf* merozoites, *Pv* merozoites, *Pf*CSP, *Pv*CSP, and *Pf*DBLα (mean increase 0.85 (95% CI: 0.57, 1.13), 0.35 (0.19, 0.50), 0.36 (0.18, 0.54), 0.18 (0.04, 0.31), 0.18 (0.01, 0.35) respectively; all p < 0.04). In contrast, antibodies targeting *Pf*VAR2CSA-expressing parasites did not show evidence of boosting in response to *P. falciparum* infection in postpartum and control women (Table 3, p = 0.89). The magnitude of boosting of antibody responses to *P. falciparum* and *P. vivax* antigens in response to a species-specific infection was similar in postpartum and control women (Table 3, p > 0.08 for interaction terms). Antibodies to *P. falciparum* merozoite antigens showed the greatest change with infection, with mean levels 0.85 (95% CI: 0.57, 1.13) higher after a *P. falciparum* infection than no infection in all women (p < 0.001).

To investigate the impact of heterologous species-infection on antibody levels, models were examined incorporating heterologous-species infection during follow-up as a time-varying variable. Infections with one species did not boost antibody responses against blood-stage antigens (Table 3, p > 0.40). However, antibody responses to the circumsporozoite proteins, *Pf*CSP and *Pv*CSP, demonstrated apparent boosting in response to heterologous infection (Table 3, p < 0.02).

## Discussion

This is the first study comparing humoral immunity to malaria over time between postpartum women and control women. Antibody responses were relatively stable in the absence of infection but more variable in those who experienced microscopically detectable infections during follow-up. Postpartum women had reduced antibody levels to *P. falciparum* and *P. vivax* antigens after delivery when compared with control women, however, postpartum levels recovered to control levels after 12 weeks. The majority of antibodies were boosted in response to species-specific infection, but there was no evidence of any boosting of antibodies against VAR2CSA-expressing parasites in the postpartum period. There were no detectable differences in the magnitude of boosting in response to infection in postpartum and control women.

The observation that antibody levels to *Plasmodium* spp. antigens increased in postpartum women over 12 weeks of follow-up suggests that humoral immunity is transitioning back to normal levels after pregnancy. This concurs with two studies in Africa that observed increased antibody levels at a single time-point (1 month and 6 weeks) postpartum compared to pregnancy^21^,^32^. Levels of circulating antibodies against merozoites and infected erythrocytes have declined during pregnancy in other studies in Africa and the Pacific^25^,^33^,^34^; the increase observed postpartum is likely reflective of a return to pre-pregnancy levels. Antibodies are preferentially transported to the fetus during pregnancy^35^, so post-pregnancy antibody levels may then increase to pre-pregnancy homeostatic levels. The haemodilution that occurs during pregnancy is followed by a period of haemoconcentration after delivery^36^; this change would be expected to result in increased antibody concentrations. Haematocrit levels in this cohort of postpartum women increased over 12 weeks postpartum^9^, however postpartum antibody levels increased even when adjusting for increasing haemconcentration suggesting that other factors play a role in changing antibody levels, such as the cessation of maternofetal antibody transfer.

Antibody responses specific to *P. falciparum* and *P. vivax* blood-stage antigens increased with homologous species-specific infections in concordance with numerous studies including a study in pregnant women from the same population^20^. We found no strong evidence of species-transcending boosting of immunity against blood-stage antigens. Antibodies against *Pf*CSP and *Pv*CSP appeared to boost in response to both species of infection, suggesting that some of the humoral response directed against CSP demonstrates species-transcending recognition, a finding that concurs with evidence from animal models^37^. We found no evidence of differences in the boosting of blood-stage or sporozoite antibodies in response to infection in postpartum women compared with control women indicating that the postpartum humoral immune system responds to *Plasmodium* spp. infection similarly to control women.

Less than 10% of women were seropositive at baseline for antibodies against VAR2CSA expressing parasites, reflecting the low endemicity of *P. falciparum* in this population; and the frequent monitoring and early treatment of malaria during pregnancy by the SMRU. Consistent with the literature^19^, antibodies against VAR2CSA were higher in women who had experienced more pregnancies. Whether VAR2CSA-specific antibody responses are maintained at adequate levels outside of pregnancy in the relative absence of VAR2CSA expressing parasites has been the matter of some uncertainty. It has also been proposed that antibodies to VAR2CSA may rapidly decay postpartum^38^. However, we found that antibodies to VAR2CSA only decayed rapidly amongst those recently exposed in pregnancy, while in other individuals VAR2CSA antibodies were relatively stable. This agrees with observed biphasic decay in antibody responses post antigen exposure^39^ and recent research investigating VAR2CSA-specific B cell memory which found that memory can be maintained for many years in the absence of antigen exposure^40^. Importantly, our study found no evidence of any boosting of antibodies against VAR2CSA-expressing parasites with infections in postpartum or control women, which would seem to support the paradigm that exposure to VAR2CSA is minimal outside of pregnancy. Our data supports the notion that antibody responses against VAR2CSA acquired in earlier pregnancies can be maintained through to future pregnancies.

Strengths of this study included the recruitment of a control group, which enabled direct comparisons between postpartum antibody responses with other women; and the weekly sampling for *Plasmodium* infection by light microscopy. However, it is probable that some women had submicroscopic infections^41^; which may have accounted for the few fluctuations observed in uninfected women. Submicroscopic carriage of parasites may also assist in the maintenance of antibody levels and differences in prevalence of submicroscopic infections between postpartum and control women may exist, though this could not be assessed in the present study. The frequent monitoring and prompt treatment of women in this study is a higher standard of treatment than many women would receive elsewhere; it is possible that untreated infections would result in a greater magnitude of antibody boosting. Additionally, this study took place in an area of low *P. vivax* and *P. falciparum* transmission, so findings may not be generalisable to areas of higher endemicity. Antibodies in this study were measured via ELISA and flow cytometry, and as yet there are no agreed upon standard or defined threshold for protection, so the clinical relevance of the reduced levels of antibodies postpartum is yet to be determined. A transient reduction in malaria-specific antibody levels would be expected to increase susceptibility to clinical malaria in some women, but as the correlates of antibody-mediated protection remain poorly defined^42^, further research is needed to assess the clinical implications of the reduction observed.

In conclusion, we observed that postpartum women had lower levels of antibodies to a variety of malaria antigens after delivery but were in the process of recovering to control levels. Despite slightly lower antibody levels, postpartum women showed no signs of impaired boosting in response to infection. Evidence from population studies in areas of higher endemicity suggests a threshold of humoral immunity is required for protective immunity^42^, a reduction in circulating levels of antibodies in postpartum women may render previously protected women susceptible in other study settings. Further studies are required to examine the immune response postpartum in different transmission settings and to determine the impact of transitioning immune responses and risk of malaria postpartum.

## Additional Information

**How to cite this article**: McLean, A. R. D. *et al*. Antibody responses to *Plasmodium falciparum* and *Plasmodium vivax* blood-stage and sporozoite antigens in the postpartum period. *Sci. Rep*. **6**, 32159; doi: 10.1038/srep32159 (2016).

## Supplementary Material

Supplementary Information

## Figures and Tables

**Table 1:** Characteristics of postpartum and non-pregnant control women.

	Postpartum (N = 201)	Controls (N = 201)	p value
**At enrolment**			
Age (years)	27.5 (22–32), [18–45.5]	28.0 (23–35), [18–50]	0.40
**Gravidity**			
Nulligravid	0 (0)	38 (18.9)	<0.001
1–2	78 (38.8)	75 (37.3)	—
3+	123 (61.2)	88 (43.8)	—
**Past behaviour**^a^			
Use of bednets	194 (93.0)	187 (96.5)	0.12
Slept outside	57 (28.4)	42 (20.9)	0.08
Worked outside	121 (60.2)	145 (72.1)	0.01
**During follow-up**			
Sera samples	4 (4–4) [1–5]	4 (3–4) [1–5]	0.07
Days between first and last sera sample	70 (70–77) [0–81]	70 (63–77) [0–84]	0.20
*** P. falciparum***			
Any infection	22 (10.8)	32 (15.8)	0.14
Number of infections	1 (1–2) [1–3]	1 (1–1.5) [1, 2]	0.53
*** P. vivax***			
Any infection	67 (33.0)	50 (24.8)	0.06
Number of infections	1 (1–2) [1–3]	1 (1–2) [1–4]	0.48
** Any** ***P. falciparum*****/*****P. vivax*** **co-infection**	1 (0.5)	4 (2.0)	0.37

Data presented as median (inter-quartile range), [minimum-maximum] or n (%). Wilcoxon rank-sum tests were performed on continuous data; chi square tests or Fisher’s exact tests (where chi square test assumptions were not met) were performed on categorical data.

^a^Behaviour prior to enrolment was reported by questionnaire.

**Table 2:** Multivariable linear-mixed effects models of *P. falciparum* and *P. vivax* antibody levels.

*P. falciparum*	Regression coefficient (95% confidence interval); p value			
	Merozoite immunity (log_2_(OD))	CSP (log_2_(OD))	DBLα (log_2_(OD))	VAR2CSA (log_2_(MFI))
Postpartum	−0.26 (−0.64, 0.12); 0.18	−0.27 (−0.47, −0.07); 0.01	−0.28 (−0.50, −0.06); 0.01	−0.18 (−0.32, −0.03); 0.02
Time (weeks) - controls^a^	−0.01 (−0.02, 0.01); 0.43	0.00 (−0.01, 0.01); 0.57	0.01 (0.00, 0.01); 0.15	0.00 (−0.01, 0.01); 0.90
Time (weeks) - postpartum^a^	0.01 (0.00, 0.02); 0.11	0.02 (0.02, 0.03); <0.001	0.02 (0.01, 0.02); <0.001	0.01 (0.00, 0.02); 0.04
History of working outdoors	0.73 (0.31, 1.14); <0.001	0.28 (0.06, 0.51); 0.01	0.26 (0.02, 0.51); 0.03	0.13 (−0.03, 0.28); 0.11
Age (per 5 years)	0.23 (0.10, 0.36); <0.001	0.05 (−0.02, 0.12); 0.13	0.02 (−0.06, 0.09); 0.69	—
Gravidity >2	—	—	—	0.29 (0.15, 0.42); <0.001
***P. vivax***				
Postpartum	−0.16 (−0.45, 0.13); 0.27	−0.27 (−0.49, −0.06); 0.01	N/A	N/A
Time (weeks) - controls^a^	0.00 (−0.01, 0.02); 0.54	0.01 (−0.01, 0.02); 0.40	N/A	N/A
Time (weeks) - postpartum^a^	0.02 (0.00, 0.03); 0.02	0.03 (0.02, 0.05); <0.001	N/A	N/A
History of working outdoors	0.43 (0.11, 0.75); 0.01	0.30 (0.06, 0.53); 0.01	N/A	N/A
Age (per 5 years)	0.16 (0.06, 0.25); 0.002	0.10 (0.03, 0.17); 0.01	N/A	N/A

Estimates of regression coefficient (95% CI) and p-value derived from linear mixed-effects modeling with adjustment for variables listed and clinic attended (Mawker Thai/Wang Pha/Walley/Mu Ler Chai).

^a^Postpartum interaction with weeks since baseline was tested using the likelihood ratio test (comparing models with and without the interaction terms) to assess if the antibody-time profiles were modified by postpartum status. For *Pf* merozoite immunity, p = 0.09; *Pf*CSP, p = 0.001; *Pf*DBLα, p = 0.13; *Pf*VAR2CSA p = 0.39; *Pv* merozoite immunity, p = 0.25; *Pv*CSP, p = 0.001.

**Table 3:** Change in antibody levels following homologous and heterologous infection.

	Regression coefficient (95% confidence interval); p value					
	*P. falciparum* infection during follow-up^a^			*P. vivax* infection during follow-up^a^		
	All women	Postpartum^b^	Controls^b^	All women	Postpartum^b^	Controls^b^
*Pf*merozoite (log_2_(OD))	0.85 (0.57, 1.13); <0.001	0.65 (0.23, 1.06); 0.002	1.02 (0.65, 1.40); <0.001	—	—	—
*Pf*CSP (log_2_(OD))	0.36 (0.18, 0.54); <0.001	0.42 (0.09, 0.75); 0.01	0.52 (0.28, 0.76); <0.001	0.14 (0.03, 0.25); 0.01	0.10 (−0.04, 0.24); 0.17	0.20 (0.04, 0.36); 0.01
*Pf*DBLα (log_2_(OD))	0.18 (0.01, 0.35); 0.03	0.20 (−0.05, 0.45); 0.12	0.17 (−0.06, 0.40); 0.14	—	—	—
*Pf*VAR2CSA (log_2_(MFI))	−0.01 (−0.19, 0.16); 0.89	−0.02 (−0.28, 0.25); 0.90	−0.01 (−0.24, 0.23); 0.94	—	—	—
*Pv*merozoite (log_2_(OD))	—	—	—	0.35 (0.19, 0.50); <0.001	0.30 (0.10, 0.51); 0.004	0.40 (0.17, 0.64); 0.001
*Pv*CSP (log_2_(OD))	0.34 (0.11, 0.57); 0.004	0.18 (−0.16, 0.52); 0.30	0.47 (0.17, 0.77); 0.002	0.18 (0.04, 0.31); 0.01	0.14 (−0.5, 0.32); 0.15	0.23 (0.02, 0.44); 0.03

Estimates of regression coefficient (95% CI) and p-value derived from linear mixed-effects modelling are presented. Models were adjusted for postpartum, clinic attended, history of working outdoors, age (except *Pf*VAR2CSA), gravidity >2 (for *Pf*VAR2CSA only) and time (weeks). The effect of heterologous species infection was assessed for each antibody response but only incorporated into the final model if there was evidence against a null effect (p < 0.05).

^a^Infection during follow-up was included as a time-varying variable with presence of infection (yes or no) at any visit prior to the corresponding antibody level measurements.

^b^Postpartum interaction with infection during follow-up was tested using the likelihood ratio test (comparing models with and without the interaction terms) to assess if the response to infection was modified by postpartum status. For *Pf*merozoite, p = 0.19; *Pf*CSP, p = 0.09; *Pf*DBLα, p = 0.89; *Pf*VAR2CSA, p = 0.97; *Pv*merozoite, p = 0.53; *Pv*CSP, p = 0.35.

## References

[b1] Dellicour S., Guerra C. A., Kuile F. O. t., Snow R. W. & Tatem A. J. Quantifying the number of pregnancies at risk of malaria in 2007: a demographic study. Plos Med. 7, e1000221 (2010). 20126256 10.1371/journal.pmed.1000221 PMC2811150

[b2] Desai M. . Review: Epidemiology and burden of malaria in pregnancy. The Lancet Infectious Diseases 7, 93–104, doi: 10.1016/s1473-3099(07)70021-x (2007). 17251080

[b3] Jamieson D. J., Theiler R. N. & Rasmussen S. A. Emerging infections and pregnancy. Emerg. Infect. Dis. 12, 1638–1643, doi: 10.3201/eid1211.060152 (2006). 17283611 PMC3372330

[b4] Fried M., Nosten F., Brockman A., Brabin B. J. & Duffy P. E. Maternal antibodies block malaria. Nature 395, 851–852, doi: 10.1038/27570 (1998). 9804416

[b5] Salanti A. . Evidence for the involvement of VAR2CSA in pregnancy-associated malaria. J. Exp. Med. 200, 1197–1204 (2004). 15520249 10.1084/jem.20041579 PMC2211857

[b6] Hviid L. The immuno-epidemiology of pregnancy-associated Plasmodium falciparum malaria: a variant surface antigen-specific perspective. Parasite Immunol. 26, 477–486, doi: 10.1111/j.0141-9838.2004.00733.x (2004). 15771683

[b7] Ramharter M. . Clinical and parasitological characteristics of puerperal malaria. J. Infect. Dis. 191, 1005–1009, doi: 10.1086/427781 (2005). 15717279

[b8] Diagne N. . Increased susceptibility to malaria during the early postpartum period. N. Engl. J. Med. 343, 598–603, doi: 10.1056/nejm200008313430901 (2000). 10965006

[b9] Boel M. E. . Malaria in the post-partum period; a prospective cohort study. Plos One 8, e57890, doi: 10.1371/journal.pone.0057890 (2013). 23516418 PMC3596341

[b10] Doolan D. L., Dobano C. & Baird J. K. Acquired immunity to malaria. Clin. Microbiol. Rev. 22, 13–36, Table of Contents, doi: 10.1128/cmr.00025-08 (2009). 19136431 PMC2620631

[b11] Osier F. H. A. . Breadth and magnitude of antibody responses to multiple Plasmodium falciparum merozoite antigens are associated with protection from clinical malaria. Infect. Immun. 76, 2240–2248 (2008). 18316390 10.1128/IAI.01585-07 PMC2346713

[b12] Richards J. S. . Association between naturally acquired antibodies to erythrocyte-binding antigens of Plasmodium falciparum and protection from malaria and high-density parasitemia. Clin. Infect. Dis. 51, e50–60, doi: 10.1086/656413 (2010). 20843207

[b13] Rono J. . Breadth of anti-merozoite antibody responses is associated with the genetic diversity of asymptomatic Plasmodium falciparum infections and protection against clinical malaria. Clin. Infect. Dis. 57, 1409–1416, doi: 10.1093/cid/cit556 (2013). 23983244 PMC3805176

[b14] Richards J. S. . Identification and Prioritization of Merozoite Antigens as Targets of Protective Human Immunity to Plasmodium falciparum Malaria for Vaccine and Biomarker Development. J. Immunol. 191, 795–809, doi: 10.4049/jimmunol.1300778 (2013). 23776179 PMC3702023

[b15] Elliott S. R. . Research priorities for the development and implementation of serological tools for malaria surveillance. F1000Prime Rep 6, 100, doi: 10.12703/p6-100 (2014). 25580254 PMC4229730

[b16] McLean A. R., Ataide R., Simpson J. A., Beeson J. G. & Fowkes F. J. Malaria and immunity during pregnancy and postpartum: a tale of two species. Parasitology, 1–17, doi: 10.1017/s0031182015000074 (2015). PMC4453920 25731914

[b17] Fried M. & Duffy P. E. Adherence of Plasmodium falciparum to Chondroitin Sulfate A in the Human Placenta. Science 272, 1502–1504 (1996). 8633247 10.1126/science.272.5267.1502

[b18] Salanti A. . Selective upregulation of a single distinctly structured var gene in chondroitin sulphate A-adhering Plasmodium falciparum involved in pregnancy-associated malaria. Mol. Microbiol. 49, 179–191 (2003). 12823820 10.1046/j.1365-2958.2003.03570.x

[b19] Ataide R., Mayor A. & Rogerson S. J. Malaria, primigravidae, and antibodies: knowledge gained and future perspectives. Trends Parasitol. 30, 85–94, doi: 10.1016/j.pt.2013.12.007 (2013). 24388420

[b20] Fowkes F. J. . New insights into acquisition, boosting and longevity of immunity to malaria in pregnant women. J. Infect. Dis. 206, 1612–1621, doi: 10.1093/infdis/jis566 (2012). 22966126 PMC3475637

[b21] Mayor A. . Immunoglobulins against the surface of Plasmodium falciparum-infected erythrocytes increase one month after delivery. Malar. J. 11, 130, doi: 10.1186/1475-2875-11-130 (2012). 22533971 PMC3423004

[b22] Fievet N. . Immune response to Plasmodium falciparum antigens in Cameroonian primigravidae: evolution after delivery and during second pregnancy. Clin. Exp. Immunol. 107, 462–467 (1997). 9067518 10.1046/j.1365-2249.1997.d01-966.x PMC1904608

[b23] Ampomah P., Stevenson L., Ofori M. F., Barfod L. & Hviid L. Kinetics of B Cell Responses to Plasmodium falciparum Erythrocyte Membrane Protein 1 in Ghanaian Women Naturally Exposed to Malaria Parasites. J. Immunol. 192, 5236–5244, doi: 10.4049/jimmunol.1400325 (2014). 24760153 PMC4025613

[b24] Cox S. E. . Rapid acquisition of isolate-specific antibodies to chondroitin sulfate A-adherent plasmodium falciparum isolates in Ghanaian primigravidae. Infect. Immun. 73, 2841–2847 (2005). 15845489 10.1128/IAI.73.5.2841-2847.2005 PMC1087373

[b25] Aitken E. H. . Antibodies to Chondroitin Sulfate A-Binding Infected Erythrocytes: Dynamics and Protection during Pregnancy in Women Receiving Intermittent Preventive Treatment. J. Infect. Dis. 201, 1316–1325, doi: 10.1086/651578 (2010). 20350189

[b26] Staalsoe T. . Acquisition and Decay of Antibodies to Pregnancy-Associated Variant Antigens on the Surface of Plasmodium falciparum-lnfected Erythrocytes That Protect against Placental Parasitemia. The Journal of Infectious Diseases 184, 618–626 (2001). 11494167 10.1086/322809

[b27] Rogerson S. J., Chaiyaroj S. C., Ng K., Reeder J. C. & Brown G. V. Chondroitin sulfate A is a cell surface receptor for Plasmodium falciparum-infected erythrocytes. The Journal Of Experimental Medicine 182, 15–20 (1995). 7790815 10.1084/jem.182.1.15 PMC2192085

[b28] Cutts J. C. . Immunological markers of Plasmodium vivax exposure and immunity: a systematic review and meta-analysis. BMC Med. 12, 150, doi: 10.1186/preaccept-1041462276129106 (2014). 25199532 PMC4172944

[b29] Fowkes F. J. I., Richards J. S., Simpson J. A. & Beeson J. G. The Relationship between Anti-merozoite Antibodies and Incidence of Plasmodium falciparum Malaria: A Systematic Review and Meta-analysis. Plos Med. 7, 1–20, doi: 10.1371/journal.pmed.1000218 (2010). PMC2808214 20098724

[b30] Hommel M. . Evaluation of the Antigenic Diversity of Placenta-Binding Plasmodium falciparum Variants and the Antibody Repertoire among Pregnant Women. Infect. Immun. 78, 1963–1978 (2010). 20160014 10.1128/IAI.01365-09 PMC2863515

[b31] Rothman K. J. No adjustments are needed for multiple comparisons. Epidemiology 1, 43–46 (1990). 2081237

[b32] Cox S. E. . Maternal vitamin A supplementation and immunity to malaria in pregnancy in Ghanaian primigravids. Trop. Med. Int. Health 10, 1286–1297, doi: 10.1111/j.1365-3156.2005.01515.x (2005). 16359410

[b33] Teo A. . Malaria preventive therapy in pregnancy and its potential impact on immunity to malaria in an area of declining transmission. Malar. J. 14, 215, doi: 10.1186/s12936-015-0736-x (2015). 26006260 PMC4449596

[b34] Campbell C. C., Martinez J. M. & Collins W. E. Seroepidemiological studies of malaria in pregnant women and newborns from coastal El Salvador. Am. J. Trop. Med. Hyg. 29, 151–157 (1980). 6989276 10.4269/ajtmh.1980.29.151

[b35] Palmeira P., Quinello C., Silveira-Lessa A. L., Zago C. A. & Carneiro-Sampaio M. IgG placental transfer in healthy and pathological pregnancies. Clin. Dev. Immunol. 2012, 985646, doi: 10.1155/2012/985646 (2012). 22235228 PMC3251916

[b36] Pritchard J. A. Changes in the blood volume during pregnancy and delivery. Anesthesiology 26, 393–399 (1965). 14313451 10.1097/00000542-196507000-00004

[b37] Yadava A., Nurmukhambetova S., Pichugin A. V. & Lumsden J. M. Cross-species immunity following immunization with a circumsporozoite protein-based vaccine for malaria. J. Infect. Dis. 205, 1456–1463, doi: 10.1093/infdis/jis220 (2012). 22457289

[b38] Hviid L., Barfod L. & Fowkes F. J. Trying to remember: immunological B cell memory to malaria. Trends Parasitol., doi: 10.1016/j.pt.2014.12.009 (2015). 25596801

[b39] White M. T. . Dynamics of the antibody response to Plasmodium falciparum infection in African children. J. Infect. Dis. 210, 1115–1122, doi: 10.1093/infdis/jiu219 (2014). 24719471

[b40] Ampomah P., Stevenson L., Ofori M. F., Barfod L. & Hviid L. B-Cell Responses to Pregnancy-Restricted and -Unrestricted Plasmodium falciparum Erythrocyte Membrane Protein 1 Antigens in Ghanaian Women Naturally Exposed to Malaria Parasites. Infect. Immun. 82, 1860–1871, doi: 10.1128/iai.01514-13 (2014). 24566620 PMC3993431

[b41] Imwong M. . The epidemiology of subclinical malaria infections in South-East Asia: findings from cross-sectional surveys in Thailand-Myanmar border areas, Cambodia, and Vietnam. Malar. J. 14, 381, doi: 10.1186/s12936-015-0906-x (2015). 26424000 PMC4590703

[b42] Stanisic D. I. . Acquisition of antibodies against Plasmodium falciparum merozoites and malaria immunity in young children: influence of age, force of infection, and magnitude of response. Infect. Immun., doi: 10.1128/iai.02398-14 (2014). PMC4294228 25422270

