# Supplementary material for: Antibody responses to Plasmodium falciparum and Plasmodium vivax blood-stage and sporozoite antigens in the postpartum period
Source: Sci Rep. 2016 Aug 25;6:32159. doi: 10.1038/srep32159 (PMC4997260; doi:10.1038/srep32159)
Supplement: Supplementary Information [file srep32159-s1.pdf]

Antibody responses to *Plasmodium falciparum* and *Plasmodium vivax* blood-stage and sporozoite antigens in the postpartum period – Supplementary Information

Alistair RD McLean[1,2], Machteld E Boel[3], Rose McGready[3,4,], Ricardo Ataide[1], Damien Drew[1], Takafumi Tsuboi[5], James G Beeson[1,6], François Nosten[3,4,], Julie A Simpson[2], Freya JI Fowkes[1,2,7]*

[1]Macfarlane Burnet Institute of Medical Research, Melbourne 3004, Australia

[2]Centre for Epidemiology and Biostatistics, Melbourne School of Population and Global Health, The University of Melbourne, Melbourne 3004, Australia

[3]Shoklo Malaria Research Unit (SMRU), Mahidol-Oxford Tropical Medicine Research Unit, Faculty of Tropical Medicine, Mahidol University, Mae Sot 63110, Thailand

[4]Centre for Tropical Medicine and Global Health, Nuffield Department of Medicine, University of Oxford, Oxford OX1 2JD, UK

[5]Division of Malaria Research, Proteo-Science Center, Ehime University, Matsuyuma 790-8577, Japan

[6]Department of Microbiology, Monash University 3800, Australia

[7]Department of Epidemiology and Preventative Medicine, Monash University 3800, Victoria, Australia

*fowkes@burnet.edu.au

Supplementary table 1. Details of antigens and sera concentrations for high throughput ELISA.

Antigen	Allele, region (amino acid position)	Expression System	Coating Concentration (μg/ml)	Sera Dilution
*Pf*EBA140$_{RIII-V}$	3D7, region III-V (746-1045)	*E. coli*	0.5	1:1000
*Pf*EBA175$_{RII}$	3D7, region II (146-713)	*P. pastoris*	0.5	1:400
*Pf*EBA175$_{RIII-V}$	3D7, region III-V (761-1045)	*E. coli*	0.5	1:400
*Pf*AMA1	3D7, whole ectodomain (25-545)	*E. coli*	0.5	1:2000
*Pf*MSP2	3D7, whole ectodomain (19-249)	*E. coli*	0.5	1:500
*Pf*Rh2	3D7, A9 (2874-3060)	*E. coli*	0.5	1:2000
*Pf*DBLα	3D7, A4 (2025-2283)	*E. coli*	0.5	1:400
*Pf*CSP	3D7, whole (323-516)	Wheat-Germ Cell-free	0.5	1:500
*Pv*AMA1	Palo Alto (25-546)	*E. coli*	0.5	1:2000
*Pv*MSP1$_{19}$	SalI, region 19 (1622-1729)	Wheat germ cell-free	0.5	1:2000
*Pv*DBP	SalI, region II-V (132-900)	Wheat germ cell-free	0.5	1:500
*Pv*CSP	i) conserved N-terminal seventy one amino acids of VK210, ii) four VK210 (Belem strain) repeat regions, iii) three VK247 (PNG strain) repeat regions, and iv) conserved C-terminal seventy amino acids of VK210.	Wheat germ cell-free	0.5	1:500

Optimisation of ELISA protocol was undertaken using a random subset of samples prior to whole cohort testing to determine the optimal concentration of antigen and concentration of sera to provide an adequate spread of OD values within the cohort.

Supplementary Table 2. Multivariable linear-mixed effects models of *P. falciparum* merozoite antibodies

	Regression coefficient (95% confidence interval); p value					
	***Pf*Rh2 (log_2(OD))**	***Pf*MSP2 (log_2(OD))**	***Pf*AMA-1 (log_2(OD))**	***Pf*EBA140$_{RIII-V}$ (log_2(OD))**	***Pf*EBA175$_{RII}$ (log_2(OD))**	***Pf*EBA175$_{RIII-V}$ (log_2(OD))**
Postpartum	-0.21 (-0.49,0.08); 0.15	-0.18 (-0.63,0.26); 0.49	-0.14 (-0.65,0.36); 0.58	-0.52 (-0.98,-0.05); 0.03	-0.09 (-0.62,0.45); 0.75	-0.42 (-0.86,0.02); 0.06
Weeks since baseline[a] (controls)	0.00 (-0.01,0.01); 0.71	0.00 (-0.02,0.03); 0.87	-0.01 (-0.03,0.01); 0.36	0.00 (-0.03,0.02); 0.91	-0.02 (-0.04,0.01); 0.15	-0.01 (-0.03,0.01); 0.37
Weeks since baseline[a] (postpartum)	0.01 (0.00, 0.02); 0.01	0.02 (0.00,0.05); 0.05	0.01 (-0.01,0.02); 0.49	0.02 (0.00,0.05); 0.18	0.02 (-0.02,0.02); 0.94	0.00 (-0.02,0.02); 0.95
History of working outdoors	0.52 (0.21,0.84); 0.001	0.65 (0.18,1.12); 0.01	1.05 (0.49,1.61); 0.001	0.85 (0.35,1.34); 0.001	0.62 (0.03,1.20); 0.04	0.65 (0.17,1.13); 0.01
Age (per 5 years)	0.11 (0.01,0.20); 0.03	0.22 (0.08,0.36); 0.003	0.28 (0.11,0.46); 0.001	0.16 (0.00,0.31); 0.05	0.43 (0.24,0.61); <0.001	0.20 (0.05,0.35); 0.01

Estimates of regression coefficient (95% CI) and p-value derived from linear mixed-effects modeling with adjustment for variables listed and clinic attended (Mawker Thai/Wang Pha/Walley/Mu Ler Chai).

[a]Postpartum interaction with weeks since baseline was tested using the likelihood ratio test (comparing models with and without the interaction terms) to assess if the antibody-time profiles were modified by postpartum status. For *Pf*Rh2 immunity, p=0.15; *Pf*MSP2, p=0.22; *Pf*AMA-1, p=0.26; *Pf*EBA140$_{RIII-V}$, p=0.18; *Pf*EBA175$_{RII}$, p=0.33; *Pf*EBA175$_{RIII-V}$, p=0.49

Supplementary Table 3. Multivariable linear-mixed effects models of *P. vivax* merozoite antibodies

	Regression coefficient (95% confidence interval); p value		
	***Pv*MSP1$_{19}$ (log$_2$(OD))**	***Pv*DBP (log$_2$(OD))**	***Pv*AMA-1 (log$_2$(OD))**
Postpartum	-0.19 (-0.54,0.16); 0.29	-0.23 (-0.46,0.00); 0.05	-0.07 (-0.57,0.42); 0.77
Weeks since baseline (controls)[a]	0.00 (-0.02,0.02); 0.76	0.00 (-0.01,0.01); 0.48	0.01 (-0.02,0.03); 0.68
Weeks since baseline (postpartum)[a]	0.01 (-0.01,0.03); 0.22	0.01 (0.00,0.02); 0.01	0.02 (0.00,0.04); 0.07
History of working outdoors	0.36 (-0.02,0.74); 0.07	0.07 (-0.18, 0.33); 0.57	0.87 (0.32,1.41); 0.002
Age (per 5 years)	0.12 (0.01,0.24); 0.04	0.08 (0.00,0.16); 0.05	0.26 (0.10,0.43); 0.002

Estimates of regression coefficient (95% CI) and p-value derived from linear mixed-effects modeling with adjustment for variables listed and clinic attended (Mawker Thai/Wang Pha/Walley/Mu Ler Chai).

[a]Postpartum interaction with weeks since baseline was tested using the likelihood ratio test (comparing models with and without the interaction terms) to assess if the antibody-time profiles were modified by postpartum status. For *Pv*MSP1$_{19}$, p=0.22; *Pv*DBP, p=0.13; *Pv*AMA1; p=0.68.

Supplementary Table 4. Multivariable linear-mixed effects models of *P. falciparum* and *P. vivax* antibody levels in postpartum women with adjustment for a documented infection during pregnancy

	Regression coefficient (95% confidence interval); p value			
P. falciparum	**Merozoite immunity (log_2(OD))**	**CSP (log_2(OD))**	**DBLα (log_2(OD))**	**VAR2CSA (log_2(MFI))**
Infection during pregnancy	1.63 (0.97,2.28); <0.001	0.47 (0.11,0.84); 0.01	0.30 (-0.11,0.71); 0.15	0.17 (-0.10,0.43); 0.23
Time (weeks)	0.01 (0.00,0.02); 0.10	0.02 (0.02,0.03); <0.001	0.02 (0.01,0.02); <0.001	0.01 (0.00,0.02); 0.05
History of working outdoors	0.48 (-0.03,0.98); 0.06	0.23 (-0.05,0.51); 0.11	0.11 (-0.20,0.43); 0.48	0.08 (-0.12,0.29); 0.43
Age (per 5 years)	0.23 (0.06,0.40); 0.01	0.13 (0.03,0.22); 0.01	0.01 (-0.09,0.12); 0.82	-
Gravidity >2	-	-	-	0.17 (-0.02,0.37); 0.08
P. vivax				
Infection during pregnancy	0.18 (-0.14,0.51); 0.26	0.08 (-0.20,0.35); 0.60	N/A	N/A
Time (weeks)	0.01 (0.00,0.02); 0.04	0.03 (0.02,0.05); <0.001	N/A	N/A
History of working outdoors	0.44 (0.13,0.76); 0.01	0.41 (0.11,0.70); 0.01	N/A	N/A
Age (per 5 years)	0.16 (0.06,0.26); 0.001	0.14 (0.04,0.24); 0.01	N/A	N/A

Estimates of regression coefficient (95% CI) and p-value derived from linear mixed-effects modeling with adjustment for variables listed and clinic attended (Mawker Thai/Wang Pha/Walley/Mu Ler Chai).

Supplementary Table 5. Change in individual merozoite antibody levels following infection

	Regression coefficient (95% confidence interval); p value					
	***P. falciparum* infection during follow-up**[a]			***P. vivax* infection during follow-up**[a]		
	**All women**	**Postpartum**[b]	**Controls**[b]	**All women**	**Postpartum**[b]	**Controls**[b]
*Pf*EBA140$_{RIII-V}$ (log_2(OD))	1.35 (0.85,1.85); <0.001	1.38 (0.63,2.12); <0.001	1.33 (0.67,1.99); <0.001	-	-	-
*Pf*EBA175$_{RII}$ (log_2(OD))	0.73 (0.23,1.22); 0.004	0.57 (-0.16,1.31); 0.13	0.85 (0.19,1.51); 0.01	-	-	-
*Pf*EBA175$_{RIII-V}$ (log_2(OD))	0.86 (0.45,1.26); <0.001	0.51 (-0.09,1.12); 0.09	1.13 (0.59,1.67); <0.001	-	-	-
*Pf*AMA-1 (log_2(OD))	1.09 (0.73,1.46); <0.001	0.87 (0.33,1.40); 0.002	1.28 (0.79,1.77); <0.001	-	-	-
*Pf*MSP2 (log_2(OD))	0.86 (0.40,1.33); <0.001	0.43 (-0.26,1.13); 0.22	1.20 (0.58,1.81); <0.001	-	-	-
*Pf*Rh2 (log_2(OD))	0.66 (0.46,0.85); <0.001	0.43 (0.14,0.72); 0.004	0.85 (0.59,1.11); <0.001	-	-	-
*Pv*AMA-1 (log_2(OD))	-	-	-	0.26 (0.02,0.50); 0.03	0.20 (-0.12,0.52); 0.21	0.34 (-0.02,0.71); 0.06
*Pv*DBP (log_2(OD))	-	-	-	0.18 (0.07,0.29); 0.001	0.16 (0.02,0.30); 0.03	0.21 (0.04,0.37); 0.02
*Pv*MSP1$_{19}$ (log_2(OD))	-	-	-	0.63 (0.40,0.86); <0.001	0.61 (0.30,0.91); <0.001	0.66 (0.31,1.01); <0.001

Estimates of regression coefficient (95% CI) and p-value derived from linear mixed-effects modelling are presented. Adjusted for postpartum, clinic attended, history of working outdoors, age (except *Pf*VAR2CSA), gravidity>2 (for *Pf*VAR2CSA only) and time (weeks). The effect of heterologous species infection was assessed for each antibody response but only incorporated into the final model if there was evidence against a null effect ($p<0.05$).

[a]Infection during follow-up was included as a time-varying variable with presence of infection (yes or no) at any visit prior to j corresponding to antibody level measurements at clinic visit j.

[b]Postpartum interaction with infection during follow-up was tested using the likelihood ratio test (comparing models with and without the interaction terms) to assess if the response to infection was modified by postpartum status. For *Pf*EBA140$_{RIII-V,}$ p=0.92; *Pf*EBA175$_{RII}$, p=0.58; *Pf*EBA175$_{RIII-V,}$ p=0.13; *Pf*AMA-1, p=0.27; *Pf*MSP2, p=0.10; *Pf*Rh2, p=0.03; *Pv*AMA-1, p=0.57; *Pv*DBP, p=0.68; *Pv*MSP1$_{19}$, p=0.82.

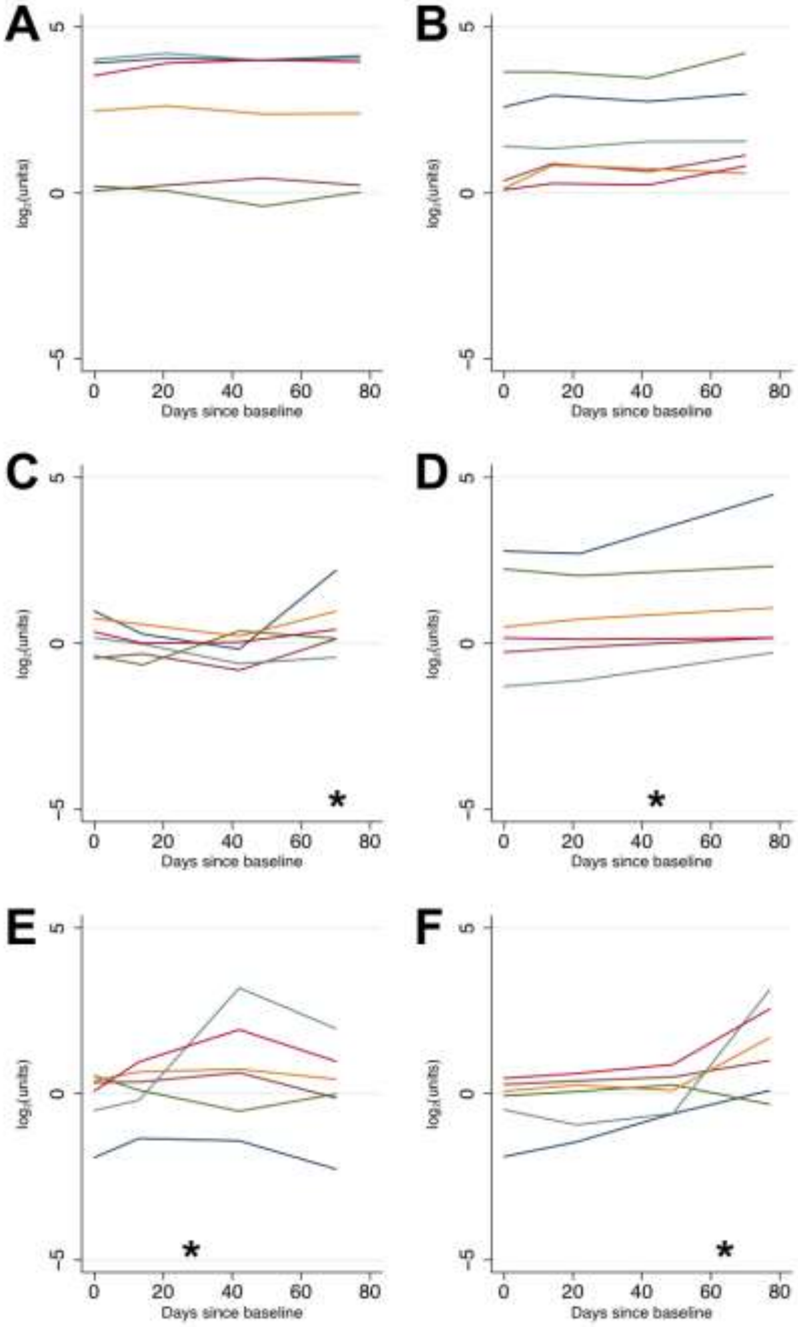

Supplementary Figure 1: Individual antibody profiles
Examples of antibody responses (log_2(MFI) for PfVAR2CSA, log_2(OD) for all other antibodies) in six individuals. A postpartum (A) and control (B) individual with no infection detected by light microscopy between the first and last antibody measurements. A postpartum (C) and control (D) individual with *P. falciparum* infection detected by light microscopy at day 70 and day 42 respectively (denoted by an asterix). A postpartum (D) and control (E) individual with *P. vivax* infection detected by light microscopy at day 28 and day 62 respectively (denoted by an asterix).

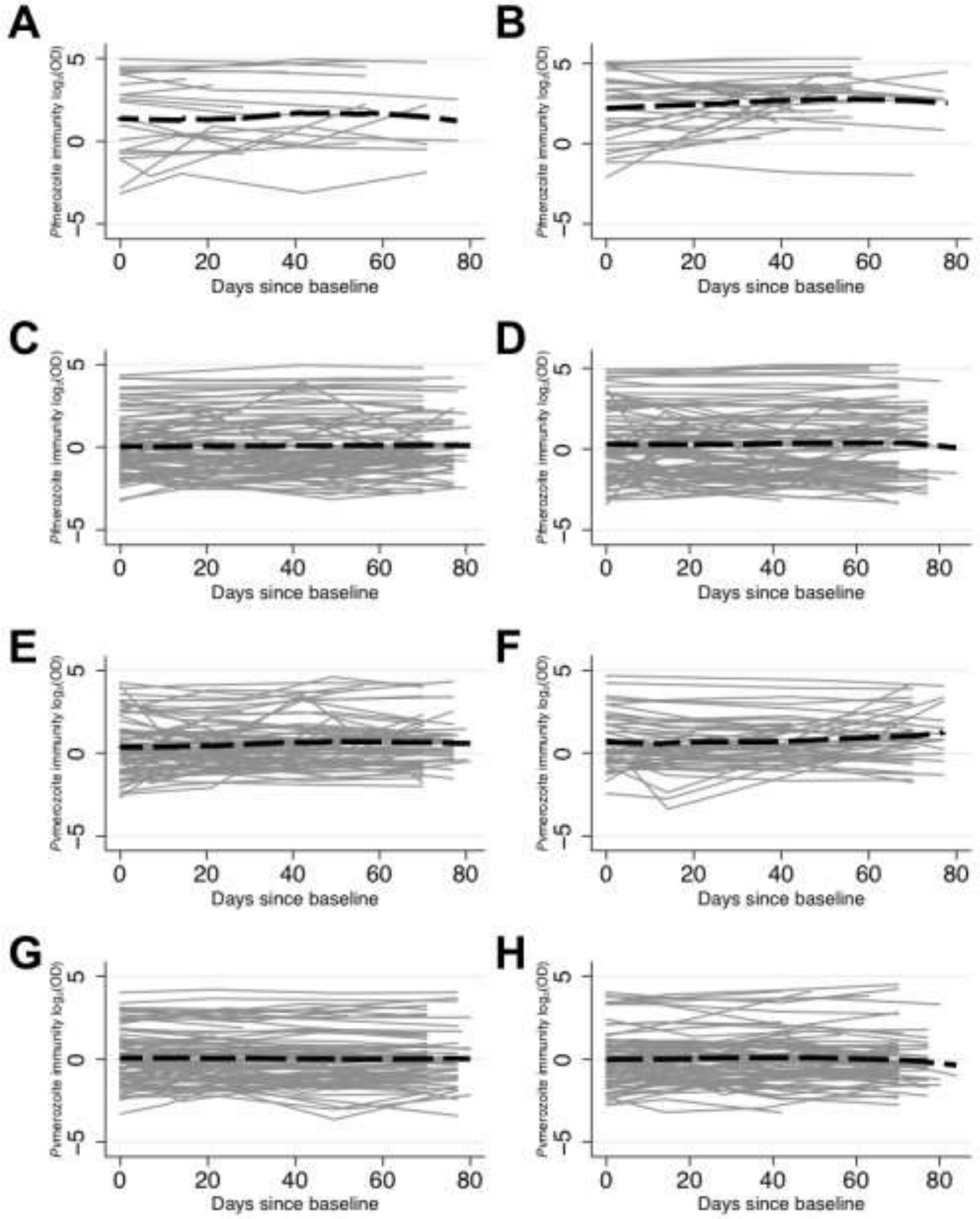

Supplementary Figure 2. Antibody levels (log$_2$ (OD)) over time (days since baseline) in postpartum and control women. Overall *P. falciparum* merozoite immunity over time in (A) postpartum women with a *P. falciparum* infection detected, (B) control women with a *P. falciparum* infection detected, (C) postpartum women with no *P. falciparum* infection detected, (D) control women with no *P. falciparum* infection detected. Overall *P. vivax* merozoite immunity over time in (E) postpartum women with a *P. vivax* infection detected, (F) control women with a *P. vivax* infection detected, (G) postpartum women with no *P. vivax* infection detected, (H) control women with no *P. vivax* infection detected. Responses for each individual are displayed in grey link plots, with an overlaid lowess (locally weighted regression) curve shown by a dashed line. For linkplots with no infection detected, 80 random women were displayed for clarity.
